# Mapping research on ICT addiction: a comprehensive review of Internet, smartphone, social media, and gaming addictions

**DOI:** 10.3389/fpsyg.2025.1578457

**Published:** 2025-05-15

**Authors:** Livia I. Andrade, Marlon Santiago Viñán-Ludeña

**Affiliations:** ^1^Department of Psychology, Universidad Técnica Particular de Loja, Loja, Ecuador; ^2^Escuela de Ingeniería, Universidad Católica del Norte, Coquimbo, Chile; ^3^Departamento de Ciencias de la Computación e Inteligencia Artificial, ETSI Informática y de Telecomunicación, CITIC-UGR, University of Granada, Granada, Spain

**Keywords:** Internet addiction, smartphone addiction, social media addiction, models, assessment tools, artificial intelligence, machine learning, deep learning

## Abstract

**Introduction:**

The use of information and communication technologies such as the Internet, smartphones, social media, and gaming has gained significant popularity in recent years. While the benefits are immense and ICTs have become essential in people's daily lives, the inappropriate use of these technologies has led to addiction, causing negative consequences in family, academic, and work environments.

**Methods:**

This study analyzes existing research related to ICT addiction (Internet, smartphone, social media, and gaming), reviewing relevant contributions. Historical trends, regions, relevance, factors, and instruments were analyzed to map out the existing research on ICT addiction.

**Results and discussion:**

The findings revealed that although the number of relevant studies has grown in recent years, there is still a lack of attention on ICT addiction and its relationship with psychological factors, social factors, physical factors, phenomenological experiences, and treatment/prevention approaches. In this regard, psychology scholars should consider appropriate methods to raise awareness about ICT addiction and emphasize the need for an in-depth understanding of the meaning, context, and practices associated with Internet, smartphone, social media, and gaming addiction.

## 1 Introduction

In recent years, interest has grown in the impact of the internet, smartphones, gaming, and social media on individuals and society. As of January 2024, more than 5.3 billion people worldwide used the internet, over 5.6 billion owned at least one mobile phone, and more than 5 billion were active on social media platforms (Kemp, 2024).

These statistics highlight the rapid global adoption of technology, which enables constant communication with loved ones, supports business transactions, provides access to information, and facilitates education through online courses from prestigious universities worldwide. Furthermore, research has indicated that smartphones play a significant role in mediating the impact of mindset on anxiety (Lai et al., 2022).

Therefore, the use of ICT technologies in tourism, marketing, politics, economics, medicine, entertainment, education, and other fields has increased year by year (Viñán-Ludeña and de Campos, 2024). However, this rise has led to the overuse or addiction to these technologies. For example, Internet addiction covers a wide variety of activities, including compulsive use of adult websites for cybersex and cyberporn, obsessive online gaming, shopping, day trading, compulsive web surfing, and so on (Widyanto and Griffiths, 2006). Personal devices such as smartphones have become essential in our lives due to their ability to keep us permanently connected to the Internet. They enable us to navigate unfamiliar places while traveling, make payments, transfers, pay for public transportation, and even find a partner through popular applications. These devices provide sociability, entertainment, information access, time management, coping strategies, and social identity maintenance (Bian and Leung, 2015; Kuss et al., 2018; Skierkowski and Wood, 2012; Panova and Carbonell, 2018). Social media, which refers to Internet-based platforms focused primarily on social interactions and user-generated content rather than licensed material from third parties, allows people to stay connected despite geographical distances and other obstacles (Kaye, 2021; Boyd and Ellison, 2007). Meanwhile, gaming is one of the largest industries in the world, offering participants the opportunity to achieve game goals, be social, and immerse themselves in the experience (Kuss et al., 2013).

Addiction is currently understood as a chronic condition characterized by pathological motivation toward certain behaviors or substance use, accompanied by impaired voluntary control. Its development and maintenance result from the interaction of biological, psychological, and social factors (Martínez-Raga et al., 2017; Ríos, 2019; West and Brown, 2013). Behavioral addictions share neuropsychological mechanisms with substance use disorders (SUDs), which is why they have been recognized as “non-substance-related disorders” by both the DSM-5 (American Psychiatric Association, 2013) and the World Health Organization (2021). These addictions are marked by intense craving, loss of control, withdrawal symptoms, and the abandonment of meaningful activities.

Currently, problematic use of the internet and mobile devices is among the most prevalent forms of behavioral addiction, producing significant functional impairments in daily life (Pedrero Pérez et al., 2017; Rodríguez et al., 2023; Tejada Garitano et al., 2023). Both substance and behavioral addictions—including problematic use of video games, social media, smartphones, and the internet—share similar neurobiological underpinnings, particularly the activation of the brain's reward system. The primary distinction lies in the nature of the reinforcing stimulus: in substance addictions, reinforcement is chemical, whereas in behavioral addictions, it is digital and social. In the case of social media, reinforcement includes notifications, posts, likes, comments, and immediate social validation. In video games, it stems from goal achievement, overcoming challenges, and obtaining virtual rewards. Problematic use of mobile phones and the internet often involves compulsive checking of notifications, continuous messaging (e.g., through WhatsApp or Telegram), frequent calls and alerts, excessive streaming or video consumption, and repetitive browsing for news or entertainment.

In this manuscript, we analyze research on behavioral addiction, focusing on the Internet, smartphones, social media, and gaming. Addictive behaviors in these domains may include online gaming, gambling, shopping, pornography consumption—including AI-enabled pornography—and instant messaging (Ioannidis et al., 2018). In addition, Artificial Intelligence (AI) is transforming various sectors of society, such as tourism, politics, and manufacturing (Viñán-Ludeña and de Campos, 2022). Psychology is no exception. Therefore, we consider AI models alongside behavioral addiction to examine how researchers worldwide are using machine learning and deep learning to investigate how, to what extent, why, and with what consequences individuals engage in problematic use of technology–ultimately leading to behavioral addiction related to the Internet, smartphones, social media, and gaming. Finally, we discuss whether AI-based models could be effectively applied to the study of behavioral addiction. Such integration may help identify gaps in the literature and inform future research agendas on ICT-related addiction.

The contributions of this review are twofold. First, it adopts a systematic and rigorous approach to summarize the empirical landscape of ICT addiction research, offering theoretical insights and proposing future research directions. Second, the findings may assist health professionals in designing targeted intervention strategies to mitigate ICT-related addiction.

## 2 Methodology

A systematic literature review (SLR) involves structured procedures for literature search, data extraction, and synthesis, often incorporating quantitative methods. Its goal is to comprehensively map the research landscape, provide an in-depth analysis of the field, and conduct a quantitative evaluation of interdisciplinary literature. This process helps identify what is known, what remains unclear, the limitations of current research, and potential opportunities for future exploration (Pickering and Byrne, 2014; Catherine et al., 2015).

This study adopts a systematic quantitative literature review rather than a narrative one. Consequently, the analysis is quantifiable, and the results are visually presented. Moreover, this approach is replicable, traceable, and supported by an explicit evidence trail that strengthens the reviewers' conclusions (Tranfield et al., 2003). It integrates various dimensions—such as geography, research scale, and methodology—to highlight key challenges and guide future research, particularly in interdisciplinary fields such as artificial intelligence (Mays et al., 2005; Yang et al., 2017). As such, it serves as an appropriate and robust method for addressing the objectives of this investigation.

**Figure 1:**
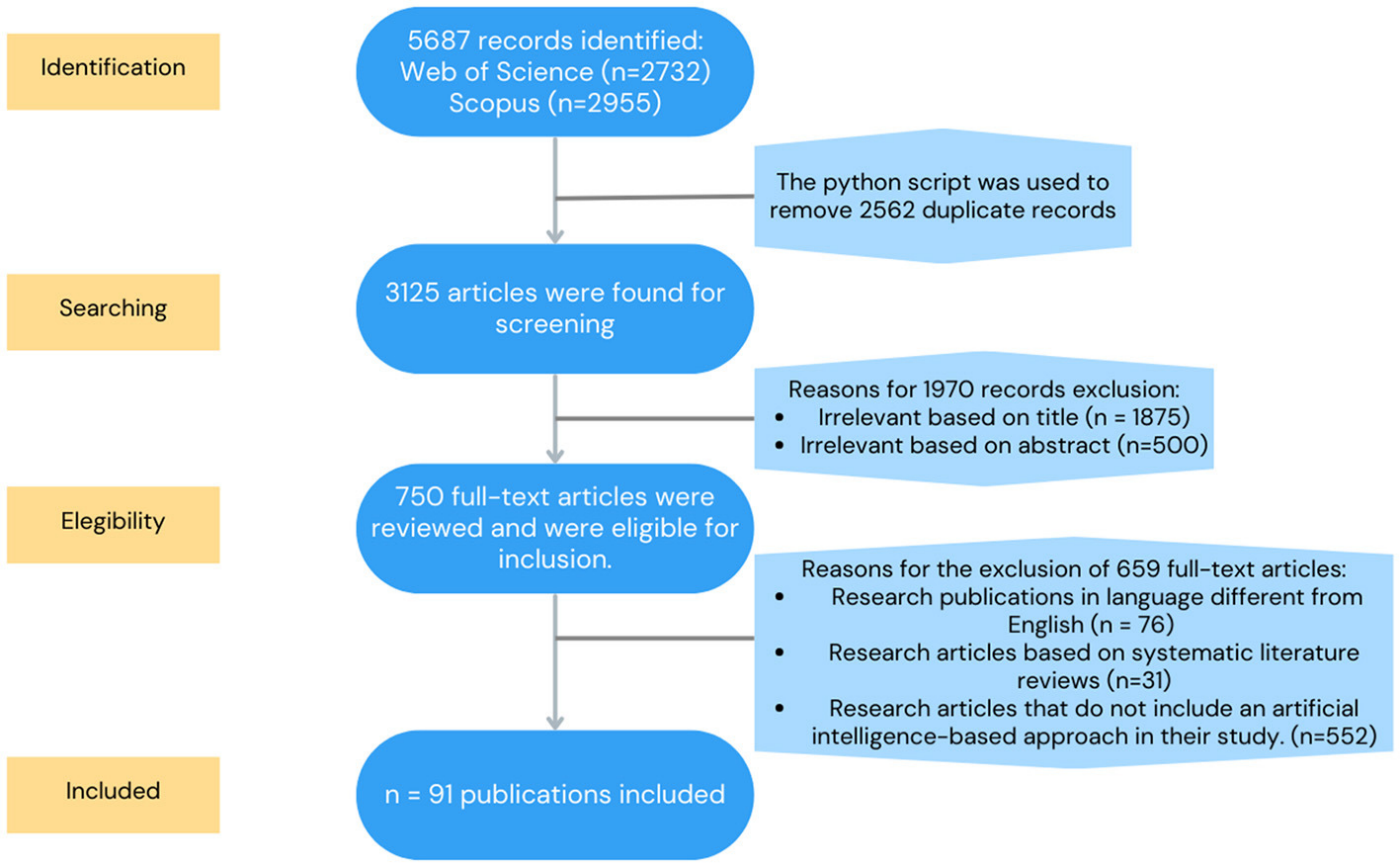
Studies selection process flowchart.

To conduct this review, we followed the Preferred Reporting Items for Systematic Reviews and Meta-Analyses (PRISMA) guidelines, a widely recognized framework in academic research. The PRISMA methodology comprises four phases: identification, screening, eligibility, and inclusion. Accordingly, the four-phase PRISMA flow diagram was applied in this study. Figure 1 illustrates the implementation of the SLR approach.

### 2.1 Eligibility criteria

Inclusion and exclusion criteria are fundamental to the research process. They help eliminate duplicate publications, low-quality studies, articles without DOIs, inaccessible sources, and studies that do not align with the objectives of the present investigation. The researchers applied the reverse snowballing technique, which begins with an initial search to identify foundational studies, followed by a more in-depth review of secondary references cited within those works. Articles were then selected and filtered according to the predefined inclusion and exclusion criteria. Furthermore, this review focused on scholarly publications that incorporated artificial intelligence-based approaches.

### 2.2 Search strategy

An extensive literature search was conducted using the Web of Science and Scopus databases, with no time restrictions applied. A broad range of search terms was employed, including Internet addiction, gaming addiction, social media addiction, smartphone addiction, and other terms related to ICT addiction. This approach aimed to capture a comprehensive selection of publications addressing the topic. Both databases were searched using the same methodology applied in previous stages. In addition, relevant journals and gray literature were manually reviewed to ensure thorough coverage.

The search was expanded to include phrases beginning with core terms related to ICT addiction, aiming to capture complementary expressions and all relevant grammatical variations. The initial search included keywords such as Internet addiction, smartphone addiction, and similar terms. Following manual inspection, additional relevant expressions were identified, including Internet abusive use, smartphone abusive use, gaming abusive use, mobile phone addiction, mobile phone abusive use, and social media abusive use.

Boolean operators “AND” and “OR” were used to construct search strings. Since digital libraries vary in how they execute search operations, queries were adapted to suit the specific syntax requirements of each database. Searches were conducted using the titles, abstracts, and keywords of peer-reviewed journal articles as the primary information sources. Articles intended for non-English-speaking audiences were excluded. A comprehensive search strategy was applied, incorporating Boolean operators and advanced syntax elements such as parentheses and quotation marks.

### 2.3 Quality evaluation

Assessing the quality and risk of bias in the selected studies is a critical step in any systematic review. This evaluation is commonly conducted using tools such as the Joanna Briggs Institute Critical Appraisal Checklist for Systematic Reviews (Joanna Briggs Institute, 2020) and the Cochrane Risk of Bias Tool (Higgins et al., 2011). These instruments help ensure that the included studies are methodologically robust and reliable.

Each primary study was evaluated for both relevance and comprehensiveness during the quality assessment process. Specifically, the quality of each selected pilot study was assessed using a predetermined set of questions. Higher scores were assigned to studies that adequately met all quality assessment criteria. In general, studies that responded to quality control questions without providing supporting evidence were not awarded points.

### 2.4 Data extraction

The extracted data were analyzed and synthesized to identify common themes, patterns, and trends related to ICT addiction and approaches involving artificial intelligence. This was carried out through thematic analysis, depending on the nature of the included studies. The initial stage of the systematic review involved extracting relevant information from each cited study. To minimize the risk of biased conclusions, two independent analysts conducted a comprehensive assessment of all data used in the analysis, which also led to additional data collection when necessary.

Subsequent data were compiled based on the research findings to provide a comprehensive overview of ICT addiction and AI-based approaches. All collected data were organized and stored in a spreadsheet. The primary articles were thoroughly screened using predetermined inclusion criteria prior to analysis.

The feasibility of the analysis was determined using both the inclusion and exclusion criteria, as well as the quality control procedures established in the review protocol. Data collection encompassed various aspects, including study methodology, data types, and geographic origin.

Following a detailed review of the data archive, each study was meticulously documented. This process included highlighting the most significant findings from the primary research, with a clear emphasis on each study's main objective. Each concept and approach was explained in detail to enhance reader comprehension.

### 2.5 Analytical approaches

In addition to conducting a systematic quantitative literature review, this study employed thematic analysis and importance assessment to evaluate the relevance and quality of the selected studies. Thematic analysis, as defined by Braun and Clarke (2006), is a flexible and foundational qualitative method for identifying, analyzing, and reporting patterns (themes) within data. It enables researchers to systematically interpret meaningful patterns in relation to specific research objectives.

Following Braun and Clarke's six-phase framework, the analysis began with familiarization with the data, followed by the generation of initial codes. Themes were then identified, reviewed, defined, and named, culminating in a comprehensive narrative synthesis. Themes were derived both inductively–from the data itself–and deductively–guided by prior research on ICT addiction and AI approaches.

This study synthesizes literature on ICT addiction that incorporates AI-based approaches. The main themes and their significance were categorized by type of addiction, including internet addiction, gaming addiction, social media addiction, and smartphone addiction. Additionally, psychological factors commonly examined in the studies–such as anxiety, depression, and impulsivity–were identified and organized thematically.

This process enabled the identification of key characteristics and the technological impact of AI in the detection and treatment of various forms of addiction. Regional differences in the application of these technologies were also examined, along with the methodological strengths and limitations reported across the studies.

## 3 Results

### 3.1 Overview

**Figure 2:**
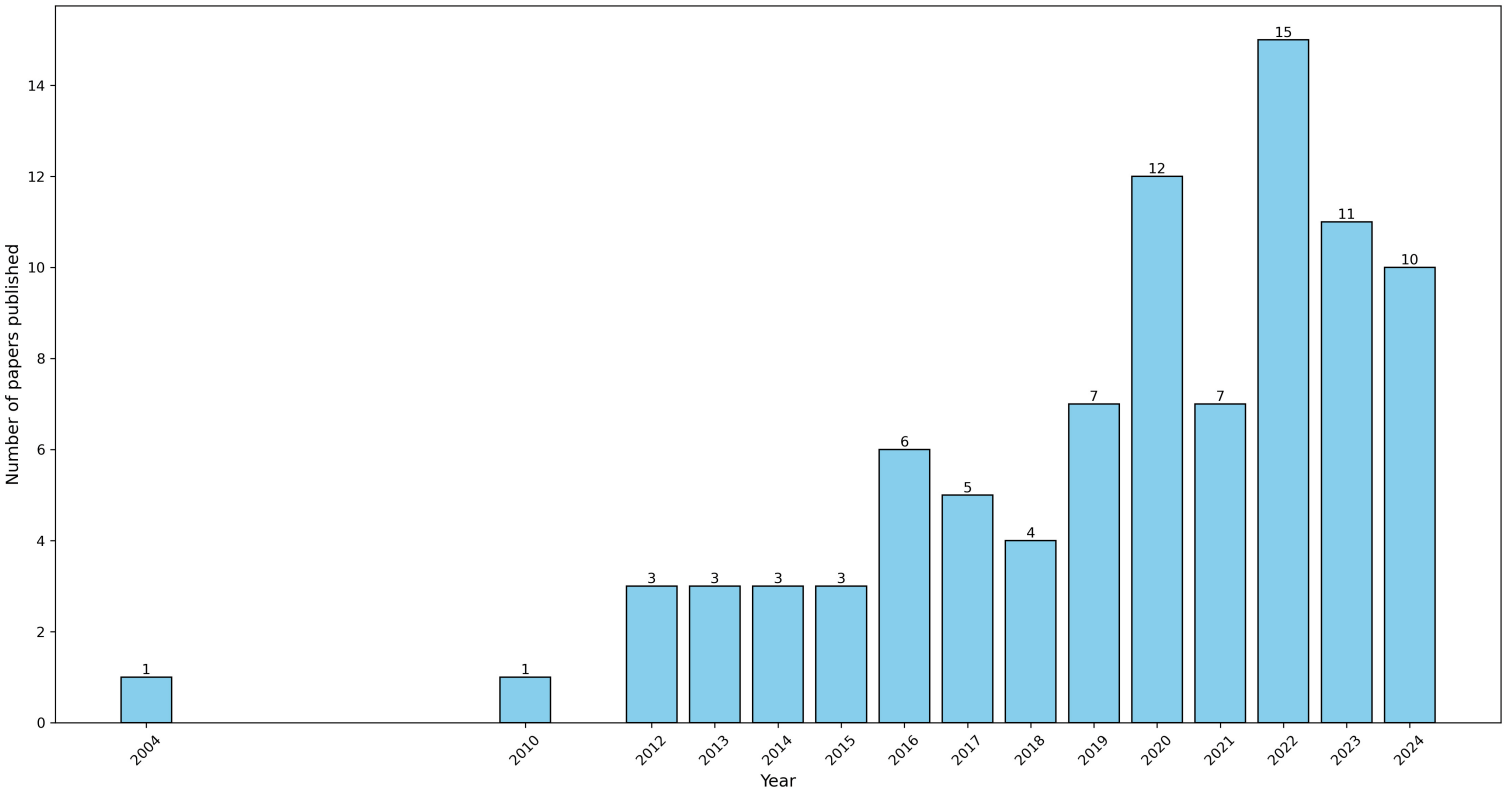
Number of published papers per year (data collected in June 2024).

Research on information technology addictions began to emerge in the early 2000s, with the earliest retrieved article published in 2004. Figure 2 illustrates the trend in publications from 2004 to June 2024. One of the most influential studies on internet addiction was conducted by Widyanto and McMurran (2004). In that study, the authors performed a systematic psychometric evaluation of the Internet Addiction Test (IAT) and identified six factors through factor analysis: salience, excessive use, neglect of work, anticipation, lack of control, and neglect of social life.

The first attempt to measure social media addiction was published by Andreassen et al. (2012). The authors developed the Bergen Facebook Addiction Scale (BFAS), an 18-item survey with three items corresponding to each of the six core components of addiction: salience, mood modification, tolerance, withdrawal, conflict, and relapse.

Interest in smartphone addiction increased significantly by 2013. One of the most notable assessments was developed by Kwon et al. (2013), who created a self-diagnostic scale to identify smartphone addiction. This tool was based on the Korean self-diagnostic program for Internet addiction (K-scale) and the specific features of smartphones. Factor analysis identified six dimensions: daily-life disturbance, positive anticipation, withdrawal, cyberspace-oriented relationships, overuse, and tolerance.

In 2014, Pontes et al. (2014) published a study on the conceptualization and measurement of Internet Gaming Disorder (IGD) as proposed in the DSM-5, and developed the IGD-20 Test. Their confirmatory factor analysis revealed a six-factor structure: salience, mood modification, tolerance, withdrawal, conflict, and relapse.

As shown in Table 1, the leading journals and their associated disciplines reflect the intersection between addiction and technology. Addictive Behaviors published the highest number of articles related to ICT addiction (n = 10, 10.99%). According to its journal statistics, Addictive Behaviors published 261 articles in 2023, which received a total of 16,259 citations. Eight articles (8.79%) were published in the International Journal of Environmental Research and Public Health.

**Table 1:** Main journals for ICT's addiction.

**Journal**	**Number of publications**	**Percentage**
Addictive Behaviors (Q1)	10	10.99
International Journal of Environmental Research And Public Health(Q2)	8	8.79
International Journal of Mental Health And Addiction (Q2)	7	7.69
Journal of Behavioral Addictions (Q1)	6	6.59
Cyberpsychology, Behavior, And Social Networking (Q1)	5	5.49
Psychiatry Research (Q1)	5	5.49
Computers in Human Behavior (Q1)	4	4.40
Frontiers in Public Health (Q2)	4	4.40
Frontiers In Psychology (Q2)	3	3.30
Plos One (Q1)	2	2.20
Asian Journal of Psychiatry (Q3)	2	2.20
Comprehensive Psychiatry (Q1)	2	2.20
Current Psychology (Q3)	2	2.20
Frontiers in Psychiatry (Q1)	2	2.20
Total	62	68.14

The other 29 articles were each published in different journals (i.e., *n* = 1 for 29 journals).

The disciplinary classifications in Table 2 were based on the introduction or scope sections of each journal. A total of 24 articles (26.37%) were classified under Public Health & Mental Health, followed closely by Addiction Studies with 23 publications (25.27%). Other categories included Cyberpsychology & Behavior (15.38%), Psychiatry (10.99%), and Miscellaneous (21.98%).

**Table 2:** Discipline of journals.

**Discipline (no of journals)**	**Number of publications**	**Percentage**
Cyberpsychology & Behavior (5)	14	15.38%
Public Health & Mental Health (16)	24	26.37%
Addiction Studies (7)	23	25.27%
Psychiatry (5)	10	10.99%
Miscellaneous (7)	20	21.98%
Total	91	100%

This table categorizes the journals into five broad disciplines and reflects the number and percentage of publications in each category.

The geographic location of research in Table 3 represents where the studies were conducted. Without taking account the studies with no specific location (*n* = 9, 9.81%), most of the studies were conducted in Asia (n=36, 39.56 %) and Europe (*n* = 28, 30.77%). Only three studies were conducted in North America (3.30%), 8 focused in Central and South America (8.79%), Cross-cultural with six studies (6.59%) and Africa with 1 paper (1.09%).

**Table 3:** Geographic distribution of research on ICT addiction.

**Geographic location of research**	**Number of publications**	**Percentage (%)**
Not Specific	9	9.81
Europe	28	30.77
Asia	36	39.56
North America	3	3.3
Central and South America	8	8.79
Africa	1	1.09
Cross-cultural	6	6.59

It is noteworthy that 39.56% of the studies analyzed originate from Asian countries. This raises the question of whether there is a genuinely higher prevalence of Information and Communication Technology (ICT) addictions in Asia, or whether this figure reflects a heightened academic interest in the topic within the region. Recent evidence suggests that certain forms of ICT addiction, particularly internet and gaming disorders, are indeed more prevalent in Asian populations compared to those in other parts of the world.

For instance, a meta-analysis by Chia et al. (2020) reported prevalence rates of 20.0% for internet addiction and 10.1% for gaming disorder in Southeast Asia—substantially higher than global averages. Similarly, Duc et al. (2024) found that 24.3% of college students in Asia exhibited signs of internet addiction, equivalent to nearly one in four students. Based on estimates from 2007 indicating 46.7 million college students in Asia, this prevalence would correspond to approximately 11.3 million individuals affected. This high burden must also be understood in light of the continent's demographic weight: as of 2025, Asia is projected to account for 58.66% of the global population (Worldometer, 2025).

In addition to population size, several sociocultural factors contribute to the elevated prevalence of ICT addiction in Asia:

- Collectivist cultural orientation: Asian societies often emphasize collectivism, which can shape online behaviors such as gaming and social media use. Higher levels of collectivism have been associated with increased internet addiction, potentially due to the cultural emphasis on group harmony and social conformity–values that may extend into digital social spaces.
- Academic pressure: Intense educational demands, particularly in East and Southeast Asia, may drive students to engage in online activities, such as gaming, as a form of escapism or stress relief (Duc et al., 2024)
- Aggressive digital marketing and accessibility: The extensive promotion of online platforms and the ubiquitous availability of digital services—especially games and social media—target adolescents and young adults, contributing to elevated usage patterns and, in some cases, problematic use.

In light of these findings, the high proportion of Asian studies in the literature not only reflects the actual burden of ICT addiction in the region but also indicates a strong academic and public health interest in addressing this growing concern. The recognition of ICT addiction as a serious societal issue has catalyzed research efforts aimed at better understanding, preventing, and mitigating its impact within these populations.

### 3.2 Main topics and relevance

#### 3.2.1 Gaming addiction

According to our results, 17 studies focused on *Gaming Addiction* (18.68%), of which 13 were psychometric analyses and 4 employed machine learning or deep learning to predict gaming addiction. One of the most commonly used questionnaires is the Internet Gaming Disorder Scale-Short Form (IGDS9-SF), which includes nine items reflecting the nine IGD criteria proposed by the APA in the DSM-5. All items are rated on a five-point Likert scale ranging from 1 (never) to 5 (very often), with higher scores indicating greater levels of disordered gaming symptoms (Maldonado-Murciano et al., 2020; Beranuy et al., 2020; Severo et al., 2020; Evren et al., 2018; Pontes et al., 2016b; Evren et al., 2020).

In contrast, the Internet Gaming Disorder Scale (IGDS) (Lemmens et al., 2015) is a more comprehensive assessment tool that includes a greater number of items designed to capture a wide range of behaviors and symptoms associated with IGD. The Internet Gaming Disorder (IGD-20) scale consists of 20 items, rated on a five-point Likert-type scale: 1 (Strongly disagree), 2 (Disagree), 3 (Neither agree nor disagree), 4 (Agree), and 5 (Strongly agree). These items assess both online and offline gaming behaviors over the past 12 months in line with IGD diagnostic criteria. Additionally, the scale evaluates six dimensions: salience, mood modification, tolerance, withdrawal symptoms, conflict, and relapse (Fuster et al., 2016; Andrade L. I. et al., 2022; Hawi and Samaha, 2017; M. et al., 2019; Ye et al., 2022; Pontes et al., 2014).

Another tool used is the Internet Gaming Disorder Self-Report for College/University Students by the ICMH-SG (ICMH-IGD scale), developed based on the IGD-20 and IGDS9-SF (Stevanovic et al., 2020). The Game Addiction Scale for Adolescents (GASA) is another instrument designed to measure computer and video game addiction. It comprises 21 items that assess seven criteria: salience, tolerance, mood modification, relapse, withdrawal, conflict, and problems (Jeroen et al., 2009).

It is also important to highlight several studies that employed alternative approaches to detect gaming addiction:

- Connectome-based predictive modeling (CPM), a machine learning method, has been used to examine potential neural mechanisms underlying addictions and other psychiatric disorders.
- To identify resting-state connections associated with IGD, the authors in Song et al. (2021) modified the core CPM algorithm using support vector machines. Functional magnetic resonance imaging (fMRI) data were collected from 72 individuals with IGD and 41 healthy control participants.
- In Wang et al. (2023), a stop-signal task (SST) was designed to assess inhibitory control in individuals with IGD using prefrontal functional near-infrared spectroscopy (fNIRS). The study involved 40 participants–24 with IGD and 16 healthy controls–and applied deep learning for classification. A 2D Convolutional Neural Network (CNN) outperformed other models and effectively predicted gaming addiction.
- Ye et al. (2022) proposed a multivoxel pattern analysis (MVPA) approach to investigate neural features of IGD using extensive neural data. Resting-state fMRI data were obtained from 402 participants with varying levels of IGD severity. Regional homogeneity (ReHo) and the amplitude of low-frequency fluctuation (ALFF) were calculated and decoded using MVPA. Results showed that both ReHo and ALFF independently and significantly predicted IGD severity.

#### 3.2.2 Internet addiction (IA)

Of the 91 studies reviewed, 38 were related to Internet addiction (41.76%), with 37 focusing on psychometric analyses and one using a machine learning approach. One of the most commonly used instruments is the Internet Addiction Test (IAT), developed by Young (1998). IAT scores have been found to positively correlate with the amount of Internet use or time spent online (Griffiths, 1998; Ferraro et al., 2006; Widyanto and McMurran, 2004), and the test has been translated and validated in numerous countries, including India, Turkey, China, Italy, Pakistan, Peru, Germany, the United States, Lebanon, Brazil, Israel, Spain, and Malaysia (Dhir et al., 2015; Kaya et al., 2016; Lai et al., 2013; Fioravanti and Casale, 2015; Ndasauka et al., 2019; Tafur-Mendoza et al., 2020; Pawlikowski et al., 2013; Jelenchick et al., 2012; Samaha et al., 2018; Brito et al., 2021; Servidio, 2017; Yaffe and Seroussi, 2019; Ali et al., 2021).

Another instrument identified in our review is the Compulsive Internet Use Scale (CIUS), developed by Meerkerk et al. (2009) to measure pathological internet use. This 14-item scale uses a 5-point Likert scale (0 = “never”, 4 = “very often”) and has been validated in multiple countries, including India, the Philippines, Turkey, Lithuania, Taiwan, Germany, Croatia, and Spain (Fernandes et al., 2021; Jusienė et al., 2023; Wartberg et al., 2014; Jovičić Burić et al., 2021; Dhir et al., 2015).

The Internet Severity and Activities Addiction Questionnaire (ISAAQ) is another tool identified in this review. It is a two-part screening instrument designed to measure the severity of Internet addiction and the amount of time spent on specific non-work and non-study-related Internet activities. Each item is rated on a 6-point Likert scale (0 = “Not at all” to 5 = “All the time”) (Omrawo et al., 2023).

The Assessment of Criteria for Specific Internet-use Disorder (ACSID-11) is a psychometric tool based on ICD-11 criteria for disorders due to addictive behaviors. It assesses specific Internet-use behaviors—such as gaming, shopping, gambling, pornography, and social media use—across 11 items grouped into three core subscales: (1) impaired control (IC), (2) increased priority (IP) given to the online activity, and (3) continuation/escalation (CE) of Internet use despite negative consequences. It also includes items assessing functional impairment in daily life (FI) and marked distress (MD) related to online activity (Müller et al., 2022; Huang et al., 2024).

The Generalized Problematic Internet Use Scale-2 (GPIUS2) consists of 15 items measuring the degree of generalized problematic Internet use. Developed by Caplan (2010), it assesses five constructs: (i) preference for online social interaction, (ii) mood regulation, (iii) cognitive preoccupation, (iv) compulsive Internet use, and (v) negative outcomes. Items are rated on a 7-point Likert scale (1 = “Strongly disagree” to 7 = “Strongly agree”) (Pontes et al., 2016a).

Another tool used to assess Internet addiction is the Internet Abusive Use Questionnaire (IAUQ), composed of 12 items rated on a 5-point Likert scale (0 = “Totally disagree” to 4 = “Totally agree”). This scale assesses three criteria: (a) excessive time spent online, (b) emotional distress when Internet use is restricted, and (c) poor control and failure to regulate usage within balanced limits (Calvo-Francés, 2016; Servidio et al., 2019).

Pontes and Griffiths (2017) developed the Internet Disorder Scale (IDS-15), and later adapted a shorter version (Pontes and Griffiths, 2016). The nine items in the short version were adapted from the DSM-5 criteria for Internet Gaming Disorder and assess salience, mood modification, tolerance, withdrawal symptoms, conflict, and relapse, all in the context of Internet use. Responses are rated on a five-point Likert scale (1 = “Never”, 5 = “Very often”) (Soraci et al., 2022).

The Internet Addiction Scale (IAS) is another self-report measure based on the seven substance dependence criteria from the DSM, along with two additional criteria proposed by Griffiths (Griffiths, 1996; Canan et al., 2010).

The Korean Scale for Internet Addiction (K-Scale), developed in South Korea, evaluates seven dimensions of addictive Internet use: “Disturbance of Adaptive Functions”, “Disturbance of Reality Testing”, “Virtual Interpersonal Relationship”, “Addictive Automatic Thought”, “Withdrawal”, “Tolerance”, and “Deviant Behavior” (Mak et al., 2017).

Finally, the Internet-Related Experiences Questionnaire (IREQ) (Fargues et al., 2009) is a 10-item, self-administered tool that assesses Internet addiction. It includes two dimensions: intrapersonal conflict and interpersonal conflict. Items are rated from 1 (not at all) to 4 (very much), with higher scores indicating greater addiction severity. This instrument has been validated in Spain (Fargues et al., 2009; Casas et al., 2013), Italy (Servidio et al., 2021), and Ecuador (Andrade et al., 2024).

A machine learning approach has also been applied to examine the influence of sociodemographic factors, Internet use intensity, types of online content accessed, activities performed, lifestyle habits, and affective temperaments on the development of problematic Internet use among adolescents (Jović et al., 2024).

#### 3.2.3 Smartphone addiction

Of the 91 papers reviewed in this study, 21 focused on smartphone addiction (23.07%), with 17 involving psychometric analyses and 5 employing machine learning methods. One of the most commonly used instruments is the Smartphone Addiction Inventory (SPAI) (Lin et al., 2014), developed to assess smartphone addiction among Taiwanese university students. It consists of 26 items rated on a four-point Likert scale from 1 (strongly disagree) to 4 (strongly agree). These items are grouped into four dimensions: Functional Impairment, Withdrawal, Compulsive Behavior, and Tolerance. The SPAI has been validated in Italy (Pavia et al., 2016), Brazil (Andrade A. L. M. et al., 2022), Turkey (Arpaci and Esgi, 2020), China (Wang et al., 2018), and Malaysia (Tan et al., 2023). A shorter version, the Smartphone Addiction Inventory - Short Form (SPAI-SF), was later developed. It includes 10 dichotomous items, with total scores ranging from 0 to 10 (Andrade et al., 2023).

The Smartphone Addiction Scale (SAS) is a self-administered questionnaire comprising 33 items rated on a six-point Likert scale. It measures six dimensions of smartphone addiction (Kwon et al., 2013): daily-life disturbance (5 items), positive anticipation (8 items), withdrawal (5 items), cyberspace-oriented relationships (7 items), overuse (4 items), and tolerance (4 items). Respondents evaluate their smartphone use characteristics from 1 (strongly disagree) to 6 (strongly agree). The SAS has been validated in Saudi Arabia (El Sayed El Keshky et al., 2022) and China (Li et al., 2024). Its short version, the Smartphone Addiction Scale - Short Version (SAS-SV), consists of 10 items covering the same dimensions and also uses a six-point Likert scale (Kwon et al., 2014). It has been validated in China (Luk et al., 2018; Cheung et al., 2019) and Italy (Servidio et al., 2023).

Manara et al. (2024) developed the Abstinence from Smartphone Scale (ABSS-10), a scale specifically designed to assess psychological responses to smartphone abstinence. The authors validated the ABSS-10 and demonstrated its relevance in the context of smartphone addiction.

Another instrument included in this review is the Smartphone Application-Based Addiction Scale (SABAS) (Csibi et al., 2018), which consists of six items rated on a six-point Likert scale from 1 (“strongly disagree”) to 6 (“strongly agree”). SABAS is based on the six components of the addiction model–salience, mood modification, tolerance, withdrawal, conflict, and relapse (Griffiths, 2005)–and has been validated in Thailand (Kamolthip et al., 2024), China (Chen et al., 2020; Leung et al., 2020), Hungary (Csibi et al., 2016), and Iran (Lin et al., 2019).

Several studies have applied machine learning approaches to smartphone addiction. Cruz et al. (2023) analyzed the use of smartphone apps for mental health and well-being (SAMHW), identifying variables that predict their use through machine learning and logistic regression. They examined how mental health indicators, smartphone usage patterns, and addiction levels relate to the use of SAMHW.

In China, Duan et al. (2021) developed a decision tree model to predict smartphone addiction among children and adolescents, based on variables such as Internet addiction, time spent on smartphones, clinical anxiety levels, fear of physical injury, and sex.

Aboujaoude et al. (2022) examined the popularity and perceived effectiveness of tools designed to monitor and limit smartphone use. They employed machine learning to identify latent user classes and their associations with demographic characteristics.

Another notable study is by Osorio et al. (2023), who developed a predictive model using machine learning to identify smartphone addiction based on the “Big Five Personality Traits (BFPT)” (Marengo et al., 2020) and the Smartphone Addiction Scale (SAS).

Finally, Elhai et al. (2020) applied supervised machine learning models to predict smartphone addiction based on demographic variables (age and sex) and psychological factors such as depression, anxiety, fear of missing out (FOMO), and rumination. Ridge, Lasso, and Elastic Net algorithms were found to be the most effective, with FOMO emerging as a particularly significant predictor.

#### 3.2.4 Social media addiction (SMA)

Fifteen papers were identified as related to social media addiction (16.48%). Of these, 13 involved psychometric analyses, and 2 employed machine learning approaches. The most commonly used instrument in this area is the Bergen Social Media Addiction Scale (BSMAS), which contains six items reflecting the core components of addiction (i.e., salience, mood modification, tolerance, withdrawal, conflict, and relapse; Griffiths, 2005). Each item assesses experiences over the past year using a five-point Likert scale ranging from 1 (very rarely) to 5 (very often). The BSMAS has been validated in Italy (Monacis et al., 2017; Andreassen et al., 2016), Iran (Lin C.-Y. et al., 2017), Taiwan (Chang et al., 2022), Turkey (Ciftci and Yildiz, 2023), Algeria (Abiddine et al., 2024), South Korea (Young, 2022), and Slovenia (Zmavc et al., 2022).

Notably, the BSMAS was adapted from the Bergen Facebook Addiction Scale (BFAS), which originally comprised 18 items based on the same six addiction components (Andreassen et al., 2012). The BFAS has been validated in Pakistan (Mahmood et al., 2022).

The 27-item Social Media Disorder Scale (SMD-27) consists of nine dimensions, each measured by three items: preoccupation, tolerance, withdrawal, persistence, escape, problems, deception, displacement, and conflict. The scale ranges from 0 to 27 (yes = 1, no = 0), with higher scores indicating greater severity of social media addiction (van den Eijnden et al., 2016; Lei et al., 2022; Watson et al., 2020).

Some researchers have employed machine learning approaches to explore the relationship between social media addiction and user health outcomes. Wong et al. (2020) examined the association between the severity of Internet Gaming Disorder (IGD), problematic social media use, sleep quality, and psychological distress among young adults. Using multiple linear regression, they found that IGDS-SF9 scores were significantly associated with psychological distress measures.

In another study, Ciftci and Yildiz (2023) investigated the relationship between social media addiction, happiness, and life satisfaction in adults. They found that as the level of social media addiction increases, life satisfaction and happiness levels decrease and according to their results, the best-performing machine learning algorithm for predicting the happiness variable was elastic net regression.

### 3.3 Psychological factors and ICT addiction

The main risk factors for technology addiction for young people are psychological vulnerability, stress, dysfunctional families and social pressure. On the other hand, protective factors are closely related to coping skills, a healthy social environment, and family support. However, in this study, we found that researchers link ICT's addiction with other psychological factors i.e. anxiety, depression, dissociative experiences, drug and alcohol use disorder, quality life, sleep quality, self-esteem and others. In the Table 4 we summarize the main factors discussed in the studies.

**Table 4:** Factors and ICT's addiction.

**Category**		**Internet addiction**	**Gaming addiction**	**Smartphone addiction**	**Social media addiction**
Mental health	Anxiety			Manara et al., 2024; Duan et al., 2021	Chang et al., 2022
	Depression	Pontes et al., 2016a		Elhai et al., 2020; Andrade et al., 2023	Wong et al., 2020; van den Eijnden et al., 2016
	Happiness				Ciftci and Yildiz, 2023
	Fear of missing out (FoMO)			Elhai et al., 2020	
	Coping Style	Boysan et al., 2017		Duan et al., 2021	
	Escapism	Fernandes et al., 2021			
	Desire thinking		Aydın et al., 2022		
	Ruminative thinking			Elhai et al., 2020	
	Attention deficit				van den Eijnden et al., 2016
	Impulsivity				van den Eijnden et al., 2016
	Obsessive-compulsive disorder	Boysan et al., 2017			
	Dissociative experiences	Boysan et al., 2017; Tudorel et al., 2019			
	Social phobia	Tudorel et al., 2019			
	Substance addictions	Sela et al., 2021			
	Hypersexual behavior	Sela et al., 2021			
	Pornography use	Sela et al., 2021			
	Self-esteem	Fernandes et al., 2021			van den Eijnden et al., 2016
	Satisfaction of life				Ciftci and Yildiz, 2023
Social factors	Quality of life	Soraci et al., 2022	Beranuy et al., 2020		
	Perceived social support	Tudorel et al., 2019			
	Academic performance	Elbilgahy et al., 2021			
	Loneliness				van den Eijnden et al., 2016
Physical factors	Obesity, physical activity				Gülü et al., 2023
	Sleep quality	Elbilgahy et al., 2021			Wong et al., 2020

As shown in the table, internet addiction appears to be the most problematic and has been associated with a wide range of factors, including anxiety and depression, coping style, escapism, obsessive-compulsive disorder, dissociative experiences, social phobia, drug use disorder, alcohol use disorder, pornography use, hypersexual behavior, quality of life, sleep quality, self-esteem, perceived social support, and academic performance.

Research on smartphone and social media addiction primarily focuses on their associations with anxiety and depression, happiness, fear of missing out (FOMO), loneliness, coping style, ruminative thinking, attention deficits, impulsivity, life satisfaction, sleep quality, and self-esteem.

Mobile phone addiction, like other behavioral addictions, is influenced by social, psychological, and physical factors. Although it is not currently recognized as a distinct diagnostic category, excessive use can significantly interfere with users' daily lives (Elamin et al., 2024; Demirci et al., 2015; Panova and Carbonell, 2018).

Finally, researchers in the field of gaming addiction often examine users' quality of life and the concept of desire thinking—defined as a perseverative focus on information, memories, and images related to a desired goal. Aydın et al. (2022) found that desire thinking is associated with Internet Gaming Disorder among adolescents and that reducing such thinking may help alleviate its symptoms. Gülü et al. (2023) explored the relationships between obesity, physical activity, and digital game addiction among adolescents, highlighting a lack of research on the connection between physical health and ICT addiction.

## 4 Discussion and conclusions

The current study systematically investigated the relevant literature on ICT addiction and artificial intelligence (AI) approaches used to evaluate and forecast this type of addiction, with a focus on historical trends, publication venues, disciplines, geographic distribution, instruments, methodologies, and key findings.

There is growing interest in this research area. Our data collection, conducted in June 2024, identified 10 publications from that year. However, the total number of studies remains limited to 91, compared to 30 articles retrieved by Lozano-Blasco et al. (2022) on internet addiction, 33 papers by Xiong et al. (2022) on smartphone addiction, 40 on gaming addiction, and 25 by Sun and Zhang (2021) on social media addiction. These earlier reviews, conducted between 2021 and 2022, highlight the need for an updated systematic review on ICT addiction.

The primary journals publishing on ICT addiction demonstrate strong interest in the topic. The most represented journals include Addictive Behaviors (10), Journal of Behavioral Addictions (6), Cyberpsychology, Behavior, and Social Networking (5), Psychiatry Research (5), Computers in Human Behavior (4), Comprehensive Psychiatry (2), and Frontiers in Psychiatry (2). In total, 34 of the 91 papers were published in Q1 journals (37.4%), suggesting increasing academic attention to technology-related addictions.

Numerous self-report instruments have been developed to assess internet addiction or problematic internet use. Young (1998) proposed two instruments: the Internet Addiction Diagnostic Questionnaire (IADQ), consisting of 8 yes/no items, and the Internet Addiction Test (IAT), comprising 20 items rated on a 6-point Likert scale. Armstrong et al. (2000) developed the Internet Related Problem Scale (IRPS), a 20-item measure using a 10-point Likert scale ranging from 1 (“not true at all”) to 10 (“extremely true”). The Online Cognition Scale (OCS), introduced by Davis et al. (2002), contains 36 items rated on a 7-point Likert scale from 1 (“absolutely disagree”) to 7 (“absolutely agree”). Meerkerk et al. (2009) developed the Compulsive Internet Use Scale (CIUS), based on substance dependence and obsessive-compulsive disorder criteria, consisting of 14 items rated on a 5-point Likert scale ranging from 0 (“never”) to 4 (“very often”). Caplan (2010) proposed the Generalized Problematic Internet Use Scale 2 (GPIUS2), which features a four-factor structure underpinning generalized problematic internet use. It includes 15 items on an 8-point Likert scale ranging from 1 (“definitely disagree”) to 8 (“definitely agree”). Finally, Demetrovics et al. (2016) introduced the Problematic Internet Use Questionnaire-Short Form (PIUQ-SF-6), which uses a three-factor structure and comprises 6 items on a 5-point Likert scale ranging from 1 (“never”) to 5 (“always/almost always”).

According to our results, research on internet addiction and its associations with mental, social, and physical factors has gained significant attention, as shown in Table 4. Several studies focus on mental health issues such as anxiety, depression, coping style, escapism, obsessive-compulsive disorder, dissociative experiences, social phobia, substance addictions, hypersexual behavior, pornography use, and self-esteem. Others address social factors including quality of life, perceived social support, and academic performance, as well as physical factors like obesity, physical activity, and sleep quality.

In addition, some studies highlight that internet addiction is associated with differences in cognitive, affective, and dispositional outcomes (Devine et al., 2022). Pjevac et al. (2024) further suggest that age, frequency of addictive substance use, purpose of internet use, and time spent online are significant predictors of internet addiction. Other contributing factors include attention-deficit/hyperactivity disorder (ADHD) symptoms and smoking addiction (Seyrek et al., 2017).

Moreover, characteristics such as high levels of parent-adolescent conflict, habitual alcohol use by siblings, perceived parental approval of adolescent substance use, and lower family functioning have been identified as predictive variables for internet addiction (Yen et al., 2007). Recent studies also suggest that factors like online khat chewing (Atalay, 2024), temperament type (Pjevac et al., 2024), poor relationships with friends and family, communication difficulties (Ahanhanzo et al., 2024), and alexithymia associated with autism spectrum disorders as strong correlates of increased susceptibility to internet addiction.

We found that the most widely used instrument for evaluating smartphone addiction is the Smartphone Addiction Inventory (SPAI). However, the first attempt to conceptualize smartphone addiction was made by Young (1998), who laid the foundation for internet addiction criteria. The K-scale was adapted from Young's original 20-item scale into a 40-item version. Subsequently, the first scale specifically designed for smartphone addiction was developed by Kwon et al. (2013), known as the Smartphone Addiction Scale (SAS). The SAS consists of 33 items divided into seven subscales: daily-life disturbance, disturbance of reality testing, positive anticipation, withdrawal, cyberspace-oriented relationships, overuse, and tolerance, with each item rated on a 6-point scale. A year later, Kwon et al. (2014) proposed a short version of the SAS with only 10 items, aimed at identifying high-risk groups in community and educational settings.

Another instrument, the Smartphone Addiction Inventory (SPAI), was developed based on the Chinese Internet Addiction Scale and the phantom vibration and ringing syndrome questionnaire. The SPAI includes four dimensions: compulsive behavior, functional impairment, withdrawal, and tolerance (Lin et al., 2014). Later, Lin Y.-H. et al. (2017) introduced the short-form SPAI-SF.

Like other behavioral addictions, mobile phone addiction is influenced by social, psychological, and physical factors. Although it is not recognized as a distinct diagnostic category, excessive use can interfere with daily life (Elamin et al., 2024; Demirci et al., 2015; Panova and Carbonell, 2018). Research has shown that problematic smartphone use is associated with physical issues such as eye strain, musculoskeletal pain, and poor sleep quality (De-Sola et al., 2017; Lee et al., 2017), as well as mental health concerns including depression, anxiety, autism, ADHD, and low self-confidence (Li et al., 2020; Elhai et al., 2016; Berg et al., 2018; Thomée, 2018).

Despite not being included in the Diagnostic and Statistical Manual of Mental Disorders (DSM-5), smartphone addiction has become a recognized social issue. Problematic behaviors related to excessive mobile phone use include: (i) using smartphones in dangerous situations; (ii) frequent disruptions to family, work, and social life, as well as physical and mental well-being; (iii) persistent urges to engage in use; (iv) dependence, tolerance, and increasing need for stimulation to achieve satisfaction; and (v) anxiety when unable to send or receive immediate responses (De-Sola Gutiérrez et al., 2016).

Major technology companies design platforms, applications, games, and AI-based systems to keep users engaged, pursuing increasingly strong stimuli that may lead to addiction. Although smartphones, tablets, and computers are valuable tools, compulsive use can interfere with work, education, and relationships. More research is needed on smartphone addiction–also known as nomophobia–to better understand its cognitive and behavioral impacts. Many studies rely on college student or child samples, which may limit the generalizability of findings. Additionally, most existing instruments are not tailored for individuals with disabilities. It is also important to note that using self-report methods may reduce the reliability of studies, as participants might overestimate their smartphone use (Montag et al., 2015; Lee et al., 2021).

The use of electroencephalography (EEG) for emotion recognition and its application to smartphone addiction is an emerging research area. Several studies have applied machine learning and deep learning models to assess smartphone dependence, highlighting the potential of these technologies. EEG-based emotion recognition remains challenging but promising, with open-source EEG databases and advanced algorithms supporting further development (Wang et al., 2023).

Researchers have emphasized that physical health indicators are essential for assessing smartphone addiction. Future studies could incorporate continuous, real-time, non-intrusive physiological measurements using electronic devices and flexible semiconductor technologies. This type of data could enhance the modeling and prediction of smartphone dependency (Seshadri et al., 2019).

Additional variables such as smartphone checking frequency and the number of notifications per app deserve more attention, despite the availability of tools for collecting smartphone usage data. Parenting neglect and poor academic performance are also important risk factors to explore. Ultimately, collaboration between psychologists and IT professionals is essential for developing tools that can map smartphone metadata to psychological indicators of addiction.

The most commonly used instrument to evaluate social media addiction is the Bergen Social Networking Addiction Scale (BSNAS), an adaptation of the Bergen Facebook Addiction Scale (BFAS). The BFAS comprises six items reflecting the core components of addiction: salience, conflict, mood modification, withdrawal, tolerance, and relapse. Researchers have found that symptoms of underlying psychiatric disorders are associated with addictive use of social networking and video gaming (Andreassen et al., 2016).

As shown in Table 4, social media addiction has attracted considerable interest from researchers, with links to various psychological and physical factors, including anxiety, depression, happiness, attention deficit, impulsivity, self-esteem, life satisfaction, loneliness, obesity, physical activity, and sleep quality. These findings align with Zhao et al. (2022), who identified impulsivity, low self-esteem, and anxiety as key risk factors for social media addiction. Similarly, Doan et al. (2022) reported a strong correlation between social media addiction and factors such as FOMO (fear of missing out) and stress. Moreover, a negative relationship between positive mental health and addictive social media use has been documented (Brailovskaia and Margraf, 2024; Jahagirdar et al., 2024; Mancin et al., 2024). Recent studies also indicate that social media addiction adversely affects users' body image awareness and appearance-related consciousness on social media platforms (Çimke and Yıldırım Gürkan, 2023).

Finally, the most commonly used instrument to assess gaming addiction is the Internet Gaming Disorder Scale (IGD-20), which includes 20 items reflecting the nine criteria of IGD outlined in the DSM-5, and incorporates the theoretical framework of the components model of addiction (i.e., salience, mood modification, tolerance, withdrawal symptoms, conflict, and relapse) (Pontes et al., 2016a).

In this study, we found that gaming addiction is associated with desire thinking and quality of life. Researchers often examine internet addiction and gaming addiction together, as users frequently spend considerable time playing online games, and the contributing factors for both conditions tend to overlap (Helga Myrseth and Borud, 2017). Additionally, Heo et al. (2024) found that attentional control significantly moderates the relationship between internet gaming addiction symptoms and attentional disengagement bias. Quancai et al. (2023) suggested that adolescents' exposure to domestic violence directly contributes to internet gaming addiction and indirectly affects it by reducing social control and self-control.

In addition, it is important to incorporate psychopathological and demographic variables to model ICT addiction using appropriate feature engineering and classification-based machine learning or deep learning algorithms to analyze and/or predict the presence or absence of ICT addiction. However, there is currently no evidence of research aimed at reducing the adverse effects of this addiction, despite the fact that early-stage corrective actions are crucial for preventing costly late interventions and long-term negative consequences. It is also worth highlighting that comparisons between online and in-person samples may be essential for fully understanding these associations, given potential differences across populations at various stages of the life course.

### 4.1 Future research

In the future, it is feasible to develop an integrated framework that leverages advancements in artificial intelligence (AI) to enhance the prevention and intervention of technology-related addictions. Such a framework could include multiple components. First, machine learning algorithms could be used to analyze behavioral data–such as screen time, application usage, and browsing patterns–to generate individualized risk profiles. Natural language processing (NLP)-based chatbots could administer psychometric assessments through conversational interfaces, dynamically adapting questions in real time using generative AI to improve diagnostic accuracy and user engagement. These systems would support early detection by tailoring screening procedures to users' responses and behavioral cues.

In subsequent stages, generative AI-powered conversational agents may simulate evidence-based practices such as motivational interviewing and cognitive-behavioral prompts, offering 24/7 support for early-stage intervention. Importantly, such systems must be capable of generating contextually relevant, empathetic dialogue that aligns with the user's emotional and linguistic patterns. While promising, it is critical to emphasize that these AI-based systems should serve as complementary tools, enhancing–not replacing–the role of qualified mental health professionals.

Artificial intelligence offers significant potential to support clinical practitioners in diagnosing disorders by processing and correlating large-scale, multimodal behavioral data–such as app usage patterns, screen time, keystroke dynamics, and smartphone sensor signals–often with greater precision than traditional clinical methods. Through the application of machine learning and deep learning algorithms, AI systems can detect patterns indicative of normative or compulsive behaviors, capturing subtle, non-obvious markers of addiction that conventional self-report instruments may fail to identify. Transformer-based models, such as BERT (Bidirectional Encoder Representations from Transformers), enhance these capabilities by analyzing user-generated content–including social media posts, chat histories, forum activity, and search queries–to identify linguistic cues associated with compulsive, impulsive, or reward-driven behaviors. Furthermore, AI enables passive, continuous, and longitudinal monitoring via smartphones and wearable devices, reducing reliance on self-reporting. This facilitates earlier detection of high-risk individuals, more accurate tracking of behavioral trajectories, and timely identification of potential relapses.

### 4.2 Limitations

The study is not without limitations. One notable limitation is the absence of a comparative analysis across regions or continents, such as Asia, the Americas, South America, or Europe. Given the influence of geography, culture, and economic context, the effects of problematic ICT use are likely to vary significantly across different populations.

Additional limitations include publication bias, limited data availability, variability in data quality, lack of granularity, and challenges in the comparability of existing studies. Moreover, with the rapid development of tools such as ChatGPT, Gemini, Perplexity, Gemma, LLaMA, Claude, etc, a new behavioral concern has emerged: generative AI user addiction. Although this phenomenon falls outside the scope of the current study, the authors acknowledge its relevance. Addiction to generative AI tools may have consequences such as diminished human creativity and reduced interpersonal interaction. As this issue is still in its early stages, it is essential for both researchers and practitioners to explore this emerging form of addiction, particularly regarding prevention and intervention strategies.

Finally, it is important to emphasize that comparing online and in-person samples may be crucial to fully understanding these associations, given potential differences between these populations at various stages of the life course.

## Data Availability

The original contributions presented in the study are included in the article/supplementary material, further inquiries can be directed to the corresponding author.
